# Cross-Database Characterization of Flavonoids and Phenolic Acids: Integrating Drug-likeness Metrics, Molecular Interactions, and Dietary Sources

**DOI:** 10.3390/molecules31040728

**Published:** 2026-02-20

**Authors:** Christmas Maria Vidal de Barros Rêgo, Zafirah Muhammad Rahman, Anna Paula Aguiar, Tatiane Fabiane Ferreira dos Santos, Sergio Senar, Luciana Aparecida Campos, Ovidiu Constantin Baltatu

**Affiliations:** 1 Center of Innovation, Technology, and Education (CITE) at Anhembi Morumbi University—Anima Institute, Sao Jose dos Campos Technology Park, Sao Jose dos Campos 12247-016, Brazil; chrisbarros1@gmail.com (C.M.V.d.B.R.); annaaguiar.galo@gmail.com (A.P.A.); tatianefabiane@hotmail.com (T.F.F.d.S.); 2 College of Medicine, Alfaisal University, Riyadh 11533, Saudi Arabia; zrahman@alfaisal.edu; 3 DrTarget, 28806 Madrid, Spain

**Keywords:** flavonoids, phenolic acids, cross-database integration, drug-likeness, QED, molecular interactions, food sources, PhytoHub, Phenol-Explorer, ChEMBL, FoodDB

## Abstract

**Background:** Flavonoids and phenolic acids are recognized for their diverse therapeutic potential, yet their translation into clinical applications remains limited by varying bioavailability and fragmented characterization across databases. A systematic integrative approach is needed to comprehensively evaluate these compounds’ drug-likeness properties based on computational metrics, molecular interactions, and dietary sources within a unified framework. **Methods:** We analyzed 954 compounds (715 flavonoids, 239 phenolic acids) by integrating data from PhytoHub, Phenol-Explorer, ChEMBL, and FoodDB databases. Drug-likeness was assessed using established metrics, including QED (Quantitative Estimate of Drug-likeness) and DataWarrior drug-likeness scores. Molecular interaction patterns were characterized through ChEMBL activity data, and food source distributions were systematically mapped across major food groups. **Results:** Drug-likeness assessment revealed complementary evaluation patterns between QED (mean = 0.48 ± 0.24) and DataWarrior scores (mean = −2.46 ± 4.38), with moderate inter-correlation (r = 0.41), indicating that each metric captures distinct aspects of molecular properties. Isoflavones demonstrated the most favorable drug-likeness profiles (mean QED: 0.62 ± 0.18). Molecular interaction analysis demonstrated significantly higher binding affinities for flavonoids (mean ChEMBL activity score: 7.26 ± 1.09) compared to phenolic acids (6.98 ± 0.94, *p* = 0.014), with flavonoids targeting a broader range of proteins (67 unique targets vs. 33 for phenolic acids). Food source mapping identified herbs and spices as the richest sources (up to 14,500 mg/kg), followed by fruits (40,490 mg/kg total) and teas (37,101 mg/kg total), with distinct compound distribution patterns across food groups. **Conclusions:** This integrative cross-database approach provides a comprehensive characterization framework for flavonoids and phenolic acids, combining established drug-likeness metrics, molecular interaction analysis, and dietary source mapping. The methodology establishes a systematic foundation for compound evaluation in drug development and nutritional research.

## 1. Introduction

Flavonoids and phenolic acids constitute major classes of plant-derived secondary metabolites extensively investigated for their wide-ranging biological activities and potential health benefits. Found in fruits, vegetables, tea, and wine, these compounds demonstrate diverse biological effects through multiple cellular mechanisms [1,2]. Their therapeutic relevance stems from their ability to modulate key cellular signaling pathways and inhibit specific enzymes such as xanthine oxidase and cyclo-oxygenase [3]. Experimental studies have indicated their potential in various health conditions, including cardiovascular diseases, neurodegenerative disorders, and metabolic conditions [4,5].

Phenolic acids and flavonoids exhibit distinct molecular interaction patterns that influence their biological activities. Previous studies have shown that flavonoids generally demonstrate interactions with a broader range of molecular targets and exhibit higher binding affinities compared to phenolic acids [6]. While flavonoids show strong binding affinity with DNA repair proteins and hormone receptors, phenolic acids exhibit focused interactions with membrane transporters, suggesting complementary roles in cellular processes [7,8,9,10].

Flavonoid supplementation has been shown to improve cardiovascular health by reducing inflammation, enhancing endothelial function, and reducing oxidative stress, thus reducing the risk of atherosclerosis [2,11]. However, the therapeutic efficacy observed in controlled settings often fails to translate effectively in clinical practice, largely due to the pharmacokinetic limitations [12]. The clinical application of flavonoids and phenolic acids faces significant challenges due to their limited bioavailability. Key limiting factors include poor aqueous solubility, extensive first-pass metabolism, and limited gastrointestinal absorption [13,14,15].

Recent advances in delivery systems, particularly nanotechnology-based approaches, show promise in addressing these limitations, but optimizing these compounds for therapeutic applications requires a systematic understanding of their molecular properties, interaction patterns with biological targets, and distribution across food sources [16,17].

Despite considerable evidence supporting the therapeutic potential of flavonoids and phenolic acids, information on these compounds is dispersed across multiple specialized databases, each providing distinct but complementary data: PhytoHub and Phenol-Explorer for compound identification and classification, ChEMBL for molecular interactions and bioactivity data, and FoodDB for dietary source distribution. Current approaches often evaluate these compounds using data from individual databases in isolation, without systematically integrating drug-likeness assessment, molecular interaction patterns, and natural source distribution. Additionally, while established drug-likeness metrics such as the Quantitative Estimate of Drug-likeness (QED) and DataWarrior drug-likeness scores provide valuable frameworks for pharmaceutical evaluation, their application to flavonoids and phenolic acids has not been systematically compared across compound subclasses.

This study aimed to address these gaps by developing an integrative cross-database characterization framework for flavonoids and phenolic acids. Specifically, we sought to: (1) systematically characterize 954 compounds across flavonoid and phenolic acid subclasses using multiple database sources; (2) evaluate drug-likeness using established metrics and assess their complementary nature; (3) analyze molecular interaction patterns and target selectivity differences between compound classes using ChEMBL bioactivity data; and (4) map compound distribution across dietary sources to identify food matrices with high flavonoid and phenolic acid content.

## 2. Methods

### 2.1. Data Collection and Molecular Dataset Preparation

PhytoHub (version 1.4) and PhenolExplorer (version 3.6) databases were selected as primary sources for compound identification and chemical characterization, yielding a dataset of 715 flavonoids and 239 phenolic acids (n = 954). All compounds were systematically characterized using standard International Chemical Identifier (InChI) keys, molecular registry numbers, and systematic nomenclature, and classified into structural classes and subclasses based on their chemical features.

The characterized compounds were cross-referenced with the ChEMBL database (version 36) to identify compounds with existing molecular registry numbers (molregno). For compounds that had InChI keys but lacked ChEMBL molecular registry numbers, structural similarity searches were conducted within ChEMBL to identify and analyze the physicochemical properties of analogous compounds.

Lastly, the FoodDB database (version 1.0) was employed to map these compounds to their dietary sources. This step facilitated the identification of food matrices and concentration ranges for the characterized flavonoids and phenolic acids.

### 2.2. Computational Analysis and Molecular Property Metrics Calculations

Molecular property analysis was performed using RDKit (version 2023.03.1) in Python (version 3.8). Using canonical SMILES representations of the chemical structures, eight molecular descriptors were calculated to assess compliance with Lipinski’s Rule of 5 (Ro5) and Veber Rules:

- Molecular weight (MW): Calculated based on atomic composition, representing the sum of atomic weights in the molecule. MW serves as a fundamental indicator of size-dependent membrane permeation and diffusion characteristics.
- LogP (Octanol-Water Partition Coefficient): Computed using RDKit’s implementation of Crippen’s method, representing the logarithm of the partition coefficient between n-octanol and water. This parameter quantifies lipophilicity, a critical determinant of membrane permeability.
- Hydrogen bond donors (HBD): Calculated as the sum of -OH and -NH groups in the molecule. HBD influences protein binding interactions and membrane permeation potential through hydrogen bonding capacity.
- Hydrogen bond acceptors (HBA): Determined by the total count of oxygen and nitrogen atoms. HBA complements HBD in characterizing a molecule’s capacity for hydrogen bonding, affecting both protein interactions and membrane passage.
- Rotatable bonds: calculated as the number of single bonds capable of free rotation (excluding terminal bonds). This parameter quantifies molecular flexibility, with fewer rotatable bonds (≤10) correlating with better oral bioavailability due to reduced entropy loss upon binding.
- Topological polar surface area (TPSA): Calculated using the Ertl et al. method as the sum of surface contributions from polar atoms (primarily oxygen and nitrogen) in Å^2^ [18]. This parameter serves as a key predictor of membrane permeability, with lower values (≤140 Å^2^) typically indicating better membrane passage.
- Aromatic rings: Determined using RDKit’s aromaticity perception algorithms. This parameter quantifies the number of aromatic systems, reflecting potential for π-π stacking interactions and molecular stability.
- Chiral centers: Identified using RDKit’s stereochemistry detection algorithms. The number of chiral centers indicates three-dimensional structural complexity and potential stereochemical influences on biological interactions.

Two sets of criteria were used to assess molecular property metrics related to drug-likeness: Lipinski’s Rule of 5 (Ro5) and Veber Rules. Ro5 dictates that a compound should ideally have a molecular weight no greater than 500 Daltons, a LogP value no greater than 5, no more than 5 hydrogen bond donors, and no more than 10 hydrogen bond acceptors. Compounds were considered Ro5 compliant if they violated no more than one of these rules. The Veber Rules stipulate that a compound should have 10 or fewer rotatable bonds and a topological polar surface area (TPSA) of 140 Å^2^ or less. Full compliance with Veber Rules required meeting both criteria.

### 2.3. Drug-likeness Assessment—QED and DataWarrior Scores

Drug-likeness was evaluated using two established computational metrics: the Quantitative Estimate of Drug-likeness (QED) score and the DataWarrior Drug-likeness score. Physicochemical parameters (molecular weight, LogP, hydrogen bond donors/acceptors, rotatable bonds, topological polar surface area) were calculated using RDKit (version 2023.03.1) in Python to support drug-likeness interpretation.

QED scores were calculated using the RDKit implementation, which combines eight molecular descriptors weighted according to the methodology described by Bickerton et al. [19]. QED calculation required complete canonical SMILES representations parseable by RDKit; compounds with missing or incompatible structural data were excluded (n = 349 of 954). DataWarrior accepts broader input formats, including InChI and molfiles, enabling drug-likeness calculation for a larger subset (n = 847).

Drug-likeness was assessed using DataWarrior software (version 5.5.0), accessing the Open Molecules database. The DataWarrior algorithm evaluates molecular substructure fragments, summing fragment contributions and normalizing by the square root of the total number of substructures [20]. Positive values indicate drug-like properties, while negative values suggest building block-like characteristics [21].

### 2.4. Molecular Interaction Analysis

Molecular interaction data were extracted from the ChEMBL database (version 33) for compounds with established molecular registry numbers (molregno). Bioactivity data were retrieved using the ChEMBL web services API, filtering for compound–protein binding activities with reported activity values.

ChEMBL activity scores, expressed as pChEMBL values (−log_10_ of the molar IC_50_, EC_50_, Ki, or Kd), were used as standardized measures of binding affinity. Only activity records meeting the following criteria were included: (1) defined target relationship (direct interaction), (2) standard activity types (IC_50_, EC_50_, Ki, Kd), and (3) activity values with specified units (nM or μM). Activity values were converted to pChEMBL scores, where higher values indicate stronger binding affinity.

Protein targets were identified using ChEMBL target identifiers and classified according to their biological function (e.g., kinases, transporters, nuclear receptors, DNA repair proteins). For each compound-target pair, the highest reported activity score was retained to avoid redundancy from multiple assays. Target diversity was quantified as the number of unique protein targets with reported binding data for each compound class.

Differences in molecular interaction strengths between flavonoids and phenolic acids were assessed using two-tailed independent samples *t*-tests. Distribution characteristics were visualized using violin plots with embedded box plots showing median, interquartile range, and 1.5× interquartile range. Statistical significance was set at *p* < 0.05.

### 2.5. Food Source Mapping and Concentration Analysis

Food source data were systematically extracted from the FoodDB database (version 1.0) using compound identifiers (InChI keys and common names) established through PhytoHub and Phenol-Explorer cross-referencing.

Compound concentrations in food matrices were retrieved and standardized to mg/kg (equivalent to mg/100 g × 10) to enable cross-food comparison. For compounds with multiple reported concentration values from different studies or analytical methods, minimum, maximum, and median concentrations were calculated. Only concentration values with defined units and quantifiable amounts (above detection limits) were included in the analysis.

Food sources were categorized into eight major food groups based on FoodDB classification and common nutritional categorization: herbs and spices, fruits, vegetables, beverages (non-tea), teas, cereals and cereal products, nuts and seeds, soy and soy products. Subclasses classifications followed botanical taxonomy (e.g., Rosaceae fruits, Brassicaceae vegetables) where applicable.

Total flavonoid content per food source was calculated as the sum of all individual compound concentrations. For each food group, the following parameters were determined: (1) total concentration (sum of all compound concentrations), (2) compound diversity (number of unique compounds detected), (3) primary compound identification (compound contributing the highest percentage to total concentration), and (4) concentration range (minimum to maximum individual food source concentrations). Hierarchical analysis stratified food sources into concentration tiers: high-content (>7000 mg/kg), medium-content (2000–7000 mg/kg), and lower-content (500–2000 mg/kg).

### 2.6. Statistical Analysis and Visualization

Statistical analyses were performed using Python’s NumPy (v1.21) and Pandas (v1.3) libraries. Distribution analyses included calculation of means, standard deviations, and compliance percentages. Kernel Density Estimation (KDE) plots were generated using Seaborn (v0.11.2) with bandwidth selection using Scott’s rule. Visualizations were created using Matplotlib (v3.4.2). Statistical computations used 64-bit floating-point precision, employing complete case analysis for handling missing values.

## 3. Results

### 3.1. Datasets Composition: Distribution and Overlap of Chemical Identifiers Across Flavonoid and Phenolic Acid Classes

**Figure 1:**
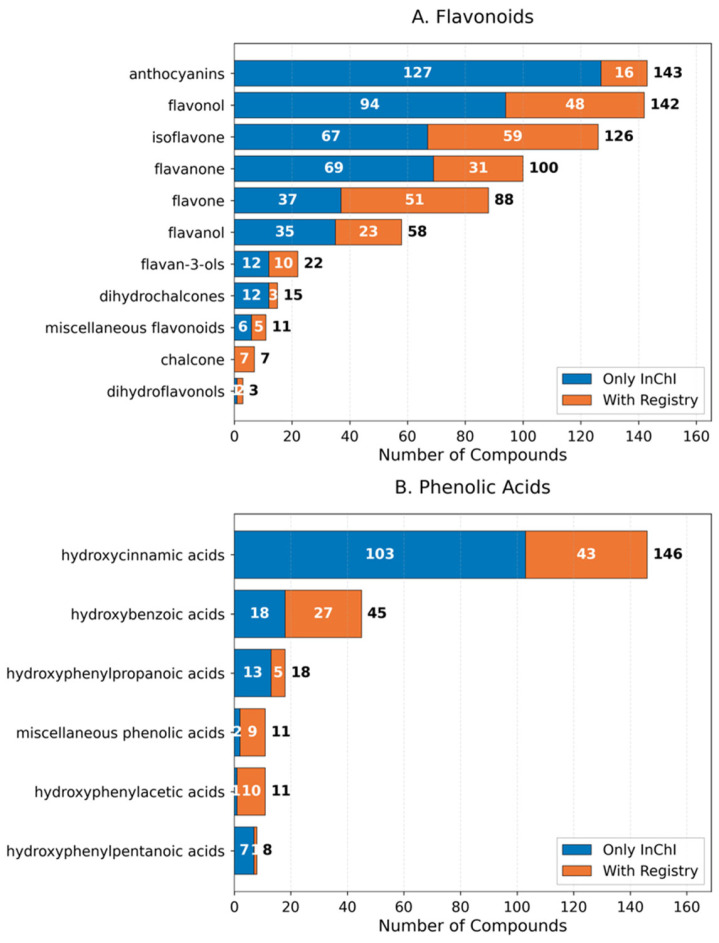
Chemical identifier distribution in phenolic compounds. The horizontal bars show the distribution of compounds identified by InChI Keys only (blue) and those with both InChI Keys and Registry Numbers (orange) for each subclass. Numbers inside bars represent compound counts for each category, while numbers at bar ends show total unique compounds. (**A**) Distribution across flavonoid subclasses. (**B**) Distribution across phenolic acid subclasses. Unique InChI Keys represent distinct chemical structures from PhytoHub and PhenolExplorer databases, while Registry Numbers correspond to compounds indexed in ChEMBL.

Chemical identifiers for flavonoids and phenolic acids were sourced from three major databases: PhytoHub, PhenolExplorer, and ChEMBL. Chemical structure identification was achieved through two standardized identifiers: International Chemical Identifiers (InChI Keys) and Molecular Registry Numbers. The distribution of chemical identifiers across phenolic compound classes revealed varying levels of structural annotation and database coverage (Figure 1).

Among flavonoids, flavonols represented the largest subclass with 324 unique compounds, of which 187 (58%) had Registry Numbers in ChEMBL. Isoflavonoids and flavones followed with 198 and 156 unique compounds, respectively, showing similar proportions of ChEMBL-indexed structures (52% and 54%). Notably, anthocyanins, despite being a smaller subclass with 89 compounds, had the highest proportion of Registry Numbers (71%). In contrast, chalcones showed the lowest database coverage, with only 38% of their 45 unique structures having Registry Numbers.

For phenolic acids, hydroxycinnamic acids emerged as the predominant subclass with 167 unique compounds, of which 103 (62%) were indexed in ChEMBL. Hydroxybenzoic acids showed a similar pattern with 89 unique compounds and 58% ChEMBL coverage. Overall, this analysis demonstrates substantial variation in the database coverage of different phenolic subclasses, with anthocyanins showing the highest representation in ChEMBL and chalcones the lowest.

During cross-database verification, three compounds—Lambertianin C (2805.91 g/mol), Sanguiin H-6 (1871.28 g/mol), and Punicalagin (1084.72 g/mol)—were identified as misclassified hydroxybenzoic acids in Phenol-Explorer and showed molecular weights that deviated substantially from the typical range of phenolic acids (mean: 357.41 ± 176.96 g/mol). Structural analysis revealed that these compounds are actually ellagitannins, a subclass of hydrolyzable tannins, rather than simple phenolic acids.

### 3.2. Drug-likeness Assessment Using Established Metrics

Drug-likeness was evaluated using two established computational metrics: the Quantitative Estimate of Drug-likeness (QED) score and the DataWarrior Drug-likeness score.

*QED Score Distribution*

**Figure 2:**
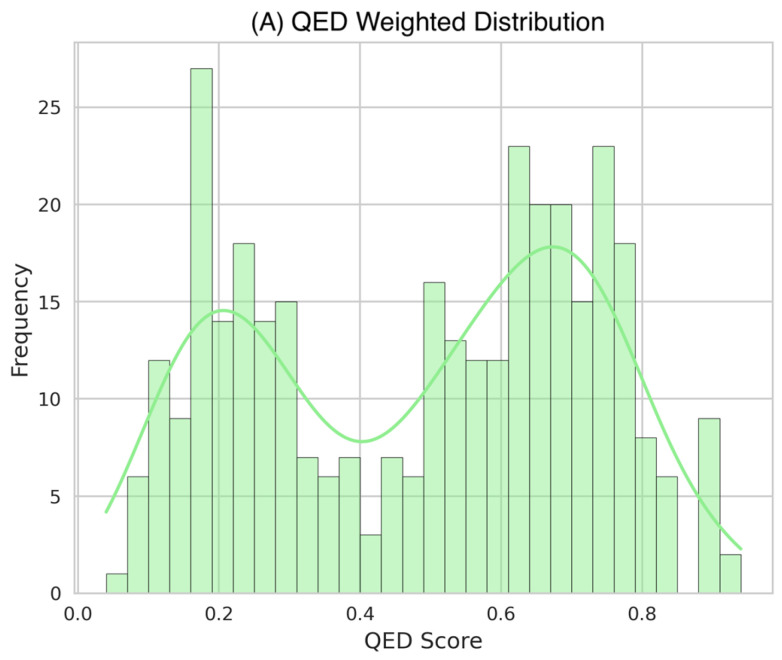

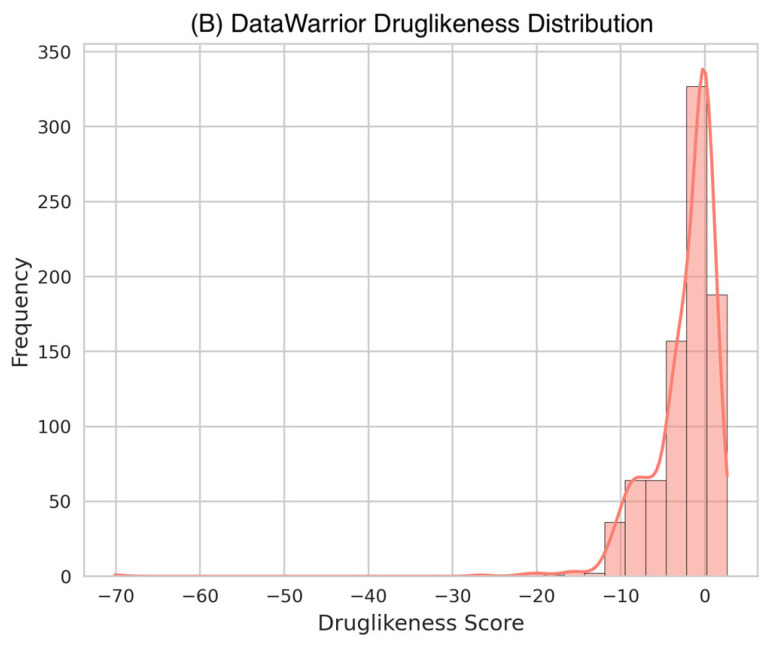
Distribution of Established Drug-likeness Metrics. Distribution plots showing the frequency of scores across the analyzed compound dataset. (**A**) QED Weighted scores show a bimodal distribution (mean = 0.48 ± 0.24) with peaks around 0.25 and 0.69, indicating two main classes of compounds with different drug-like characteristics (n = 349). (**B**) DataWarrior Drug-likeness scores exhibit a wide range (mean = −2.46 ± 4.38) with a left-skewed pattern, reflecting varying degrees of predicted drug-like properties (n = 847). All distributions include kernel density estimation curves (solid lines) to highlight the underlying probability density of the scores.

QED scores were calculated for 349 compounds with complete structural data. The analysis revealed a distinctive bimodal distribution with a mean of 0.48 ± 0.24 (Figure 2A). QED scores showed a slight negative skew (skewness = −0.155), with an interquartile range from 0.25 to 0.69, suggesting the presence of two major compound populations with distinct drug-like characteristics. This bimodality likely reflects the structural diversity within the dataset, particularly in terms of molecular complexity and pharmaceutical relevance.

The top-performing compounds by QED score included Sativanone (0.940), Dihydroformononetin (0.910), and Violanone (0.900), approaching the theoretical maximum QED value of 1.0. The median QED score of 0.530 suggests that a substantial portion of the analyzed compounds possess favorable drug-like properties according to this metric.

*DataWarrior Drug-Likeness Score Distribution*

DataWarrior drug-likeness scores were determined for 847 compounds, displaying a notably left-skewed distribution (skewness = −5.364) with a mean of −2.46 ± 4.38 (Figure 2B). The broad range of scores, from a minimum of −70.14 to a maximum of 2.65, with a median of −0.98, highlights the heterogeneous nature of the compound set. Positive values indicate drug-like properties, while negative values suggest building block-like characteristics.

Notably, 25% of the compounds showed favorable drug-likeness scores above 0.05, identifying a substantial subset of compounds with favorable computed profiles suggestive of drug-like characteristics. Modified flavonoids demonstrated superior drug-likeness properties, with 3′-O-Methyl-(-)-epicatechin 4′-O-sulfate achieving the highest score (2.648), followed by Genistein-7-O-glucuronide-4′-sulfate (2.562). This suggests that specific structural modifications, particularly sulfation and glucuronidation, may enhance the drug-like properties of flavonoid compounds.

### 3.3. Correlation Analysis of Drug-likeness Metrics

**Figure 3:**
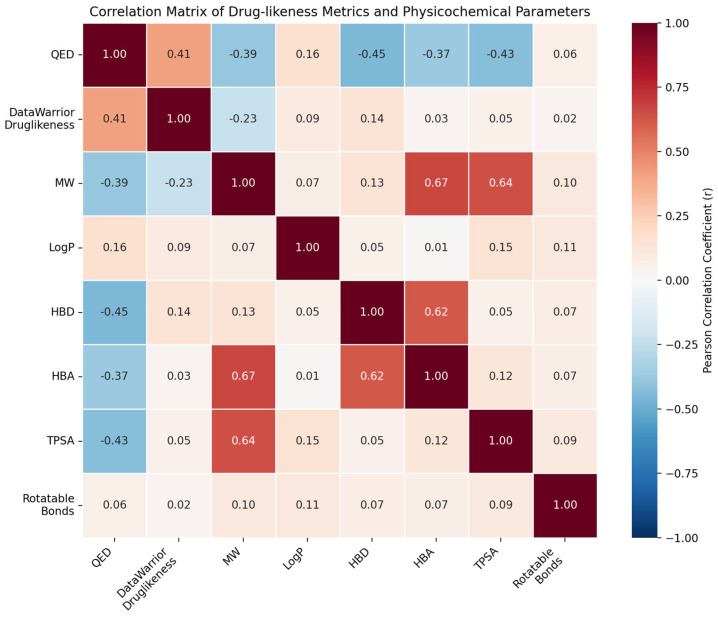
Correlation Matrix of Drug-Likeness Scoring Systems. The matrix displays pairwise Pearson correlation coefficients between drug-likeness metrics: QED (Quantitative Estimate of Drug-likeness) and DataWarrior Drug-likeness scores, along with key Lipinski parameters (MW, LogP, HBD, HBA). Correlation coefficients range from −1 (perfect negative correlation, red) to +1 (perfect positive correlation, blue), with 0 indicating no correlation (white). The moderate correlation coefficient (r = 0.45) between QED and DataWarrior scores suggests that each metric captures distinct and complementary aspects of molecular properties, validating their combined use in compound evaluation.

The correlation matrix (Figure 3) reveals distinct relationships between drug-likeness scores and molecular descriptors across the compound library. QED and DataWarrior drug-likeness scores showed moderate positive correlation (r = 0.41). This correlation was calculated using the 349 compounds with both QED and DataWarrior scores available. The subset with complete data for both metrics may be biased toward structurally simpler compounds, and correlation results should be interpreted within this context. QED exhibited negative correlations with molecular weight (r = −0.39), hydrogen bond donors (r = −0.45), hydrogen bond acceptors (r = −0.37), and topological polar surface area (r = −0.43). Strong positive correlations were observed between molecular weight and both hydrogen bond acceptors (r = 0.67) and TPSA (r = 0.64). Hydrogen bond donors and acceptors were also strongly correlated (r = 0.62).

### 3.4. Compound Classification by Drug-likeness Profiles

Analysis of drug-likeness metrics across compound subclasses revealed distinct patterns (Table 1). Compounds were stratified based on their QED and DataWarrior scores to identify subclasses with favorable pharmaceutical profiles.

Isoflavones demonstrated the most favorable drug-likeness profiles, with the highest mean QED score (0.62 ± 0.18) and positive DataWarrior scores (0.42 ± 2.1). This subclass also showed the best compliance with Lipinski parameters, with moderate molecular weight (364.4 Da), acceptable LogP (1.60), and hydrogen bonding capacity within optimal ranges. In contrast, flavan-3-ols and flavonols showed lower drug-likeness scores, primarily due to higher molecular weights, excessive hydrogen bond donors, and elevated polar surface areas.

Table 2 presents the top 10 compounds identified by each established drug-likeness metric, including their 2D chemical structures to facilitate rapid structural assessment. The QED analysis identified isoflavones and flavanones as top performers, with sativanone achieving the highest score (0.940). These compounds share structural features conducive to oral bioavailability: moderate molecular weight, balanced lipophilicity, and limited hydrogen bonding capacity. The DataWarrior analysis highlighted the importance of specific structural modifications. Sulfated and glucuronidated flavonoid conjugates dominated the top rankings, suggesting that these phase II metabolite forms may possess enhanced drug-like properties compared to their parent aglycones.

The QED analysis identified isoflavones and flavanones as top performers, with sativanone achieving the highest score (0.940). These compounds share structural features conducive to oral bioavailability: moderate molecular weight, balanced lipophilicity, and limited hydrogen bonding capacity. The DataWarrior analysis highlighted the importance of specific structural modifications. Sulfated and glucuronidated flavonoid conjugates dominated the top rankings, suggesting that these phase II metabolite forms may possess enhanced drug-like properties compared to their parent aglycones.

### 3.5. Differential Molecular Interaction Patterns Between Flavonoids and Phenolic Acids

**Figure 4:**
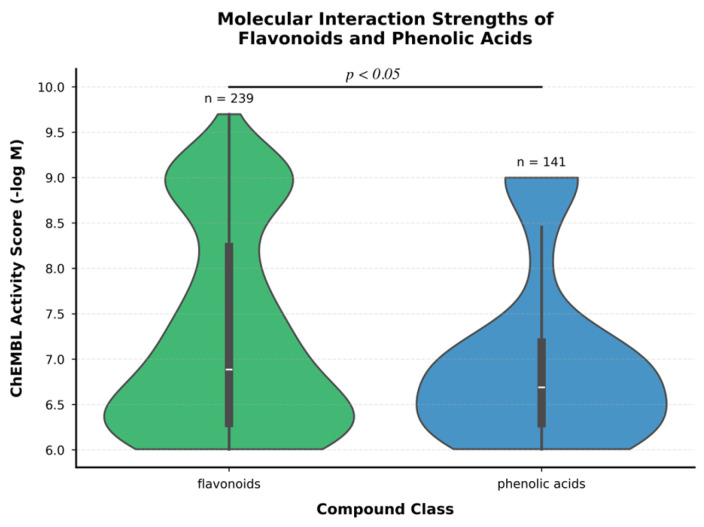
Molecular Interaction Strength Distribution of Flavonoids and Phenolic Acids. Violin plots showing the distribution of ChEMBL activity scores (−log M) for flavonoids (n = 239) and phenolic acids (n = 141). The broader distribution and higher median for flavonoids indicate greater target diversity and binding strength, which may underlie their pleiotropic biological effects. Internal box plots display the median (horizontal line), interquartile range (box), and 1.5× interquartile range (whiskers). The statistically significant difference (*p* < 0.05) supports distinct pharmacological profiles warranting class-specific development approaches.

Analysis of molecular interaction strengths revealed distinct binding patterns between flavonoids and phenolic acids across their protein targets (Supplementary sheet ‘molTargetDiseaseEdges’ in Excel file ‘flavonoidTables Manuscript.xlsx’). Flavonoids exhibited significantly higher overall interaction strengths (mean ChEMBL activity score: 7.26 ± 1.09) compared to phenolic acids (6.98 ± 0.94; *p* = 0.014, two-tailed *t*-test). The distribution of activity scores showed greater variability for flavonoids (range: 6.01–9.70) than phenolic acids (range: 6.01–9.00), suggesting more diverse interaction patterns. Flavonoids demonstrated exceptional binding affinity with DNA repair proteins, particularly the Bloom syndrome protein (activity score: 9.70), and the thyroid hormone receptor beta-1 (activity score: 9.22). In contrast, phenolic acids showed more concentrated interaction patterns, with their highest affinities consistently observed for membrane transporters, specifically the SLCO1B1 and SLCO1B3 proteins (activity scores: 9.00). This specialized targeting of transport proteins by phenolic acids suggests a potential role in cellular uptake and distribution mechanisms. The broader distribution of flavonoid interaction strengths, evidenced by the wider violin plot (Figure 4), indicates greater molecular promiscuity compared to phenolic acids. This characteristic may explain their diverse biological effects reported in previous studies. The higher median activity score for flavonoids (6.89) compared to phenolic acids (6.69) further supports their broader interaction potential with biological targets.

Flavonoids demonstrated interactions with a broader range of targets (67 unique targets) compared to phenolic acids (33 targets), with higher overall binding affinities across their target spectrum (Table 3).

### 3.6. Distribution of Flavonoids Across Food Sources

**Figure 5:**
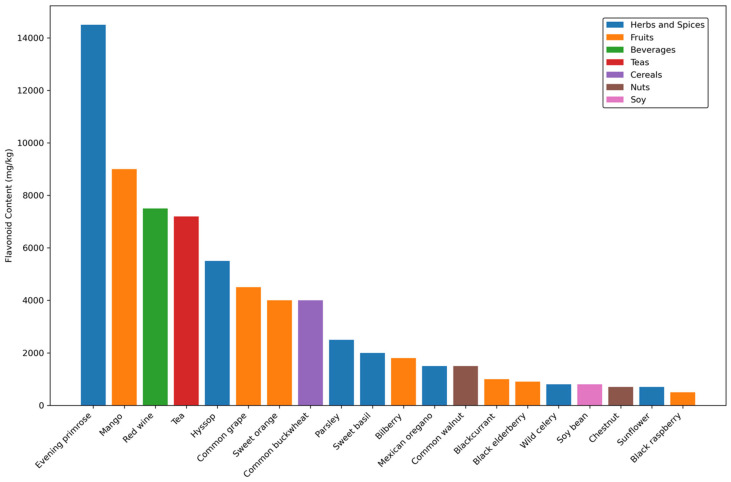
Distribution of total flavonoid content across food sources. Bar graph illustrating flavonoid concentrations (mg/kg) in the top 20 food sources, categorized by food groups: herbs and spices (blue), fruits (orange), beverages (green), teas (red), cereals (purple), nuts (brown), and soy (pink). This visualization identifies priority dietary sources for flavonoid intake, with herbs/spices and certain fruits emerging as the most concentrated sources, informing dietary recommendations for maximizing flavonoid consumption. Concentration values represent single reported measurements from FoodDB; error bars are not included as multiple independent measurements were unavailable for most sources. Subclass composition for each food group is detailed in Figure 6.

**Figure 6:**
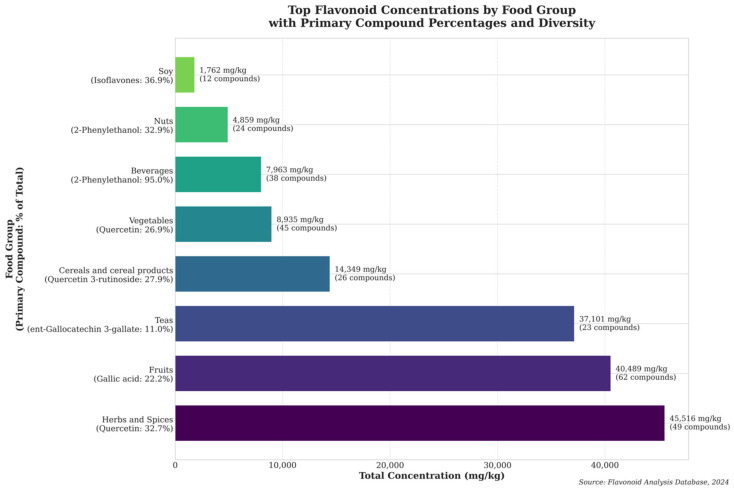
Distribution and Composition of Flavonoids Across Major Food Groups. Total flavonoid concentrations (mg/kg) are shown as horizontal bars for the eight major food groups, with primary compound and percentage contribution indicated in parentheses. Compound diversity (number at bar end) reveals that fruits offer the broadest flavonoid spectrum (62 compounds) despite lower total concentration than herbs/spices, suggesting different food groups may be optimal for either concentrated intake or diverse compound exposure. This distinction informs targeted dietary strategies based on specific health objectives.

The analysis of flavonoid content across diverse food sources revealed distinctive patterns in total concentrations, with values ranging from 500 to 14,500 mg/kg (Figure 5; Supplementary sheet ‘parentFlavonoidsInFoods’ in Excel file ‘flavonoidTables Manuscript.xlsx’). These concentration values represent chemical abundance in food matrices, not bioavailable or nutritionally efficacious amounts; actual nutritional impact depends on bioaccessibility, absorption efficiency, and metabolic fate, which vary substantially among compounds and individuals. Evening primrose emerged as the predominant source, containing approximately 14,500 mg/kg of total flavonoids, substantially higher than other analyzed sources. Concentration values represent single measurements reported in FoodDB; the relationship between total concentrations and flavonoid subclass composition across food groups is presented in Figure 6. This was followed by mango (9000 mg/kg) and red wine (7500 mg/kg), establishing the top tier of flavonoid-rich sources. Tea varieties demonstrated consistently high concentrations at approximately 7200 mg/kg, indicating their significance as a reliable flavonoid source.

The distribution of flavonoid content demonstrated clear categorical patterns. Herbs and spices emerged as particularly rich sources, with evening primrose, hyssop (5500 mg/kg), and parsley (2500 mg/kg) representing the highest concentrations within this category. Fruit sources showed notable variation, with mango, common grape (4500 mg/kg), and sweet orange (4000 mg/kg) containing the highest concentrations among fruits. Beverages, particularly red wine and tea, constituted another significant category, consistently showing high flavonoid content above 7000 mg/kg.

A hierarchical analysis revealed three distinct tiers of flavonoid content. The high-content tier (>7000 mg/kg) included evening primrose, mango, red wine, and tea varieties. The medium-content tier (2000–7000 mg/kg) encompassed several herbs and fruits, including hyssop, common grape, sweet orange, and parsley. The lower-content tier (500–2000 mg/kg) included a diverse range of sources such as bilberry, Mexican oregano, common walnut, and black raspberry, demonstrating that even foods with relatively lower concentrations can contribute meaningfully to dietary flavonoid intake.

Notably, certain food categories showed consistent patterns within their groups. Herbs and spices generally maintained high concentrations, while fruits demonstrated more variable content. Cereals and nuts, represented by common buckwheat (4000 mg/kg) and common walnut (1500 mg/kg, respectively, showed moderate flavonoid content. These findings suggest that optimal dietary flavonoid intake might be achieved through a diverse diet incorporating multiple food categories, with particular emphasis on herbs, spices, and specific fruits.

### 3.7. Distribution and Composition of Bioactive Compounds Across Food Groups

Analysis of flavonoid distribution revealed distinct patterns of compound accumulation and diversity across major food groups (Figure 6).

Herbs and spices emerged as the richest source of flavonoids, with a total concentration of 45,516 mg/kg, characterized by a predominance of quercetin (32.74%) and substantial compound diversity (49 unique compounds). This was followed by fruits, which exhibited the second-highest total concentration (40,490 mg/kg) and demonstrated the greatest compound diversity with 62 unique flavonoids, primarily composed of gallic acid (22.23%).

Teas represented the third most concentrated source (37,101 mg/kg), showing a more specialized profile dominated by ent-gallocatechin 3-gallate (11.02%). Notably, cereals and cereal products contained significant flavonoid levels (14,350 mg/kg), with quercetin 3-rutinoside as the primary compound (27.88%). Vegetables, while showing moderate total concentrations (8935 mg/kg), maintained considerable compound diversity with 45 unique flavonoids. Beverages displayed the highest compound specificity among all food groups, with 2-phenylethanol constituting 95.04% of total flavonoids (7963 mg/kg). Nuts and soy products, while containing lower total concentrations (4859 and 1762 mg/kg, respectively), showed distinct compound profiles, with nuts rich in 2-phenylethanol (32.93%) and soy characterized by isoflavones (36.91%).

These findings highlight the differential accumulation patterns of flavonoids across food groups, revealing both specialized (e.g., beverages) and diverse (e.g., fruits, herbs and spices) compound profiles. The data suggest that dietary sources of flavonoids vary not only in total concentration but also in compound composition and diversity, which may have implications for their biological activities and potential health benefits.

## 4. Discussion

The analysis of 954 phenolic compounds (715 flavonoids, 239 phenolic acids) integrated data from multiple databases to examine their drug-likeness characteristics, molecular interactions, and food source distribution. This integrative approach addresses the fragmentation of phytochemical data across specialized databases and provides a comprehensive characterization framework for these bioactive compounds.

Chemical identifier analysis revealed differential representation across subclasses, with anthocyanins showing 71% representation in ChEMBL compared to chalcones (38%), reflecting database cataloging patterns and research prioritization rather than inherent biological significance. For phenolic acids, hydroxycinnamic acids showed 62% ChEMBL coverage, consistent with their extensive documentation in nutritional research literature [22,23]. These coverage variations highlight the importance of integrating multiple databases to achieve comprehensive compound characterization, as reliance on a single source may introduce systematic biases in compound evaluation.

Structural analysis identified three high-molecular-weight compounds (Lambertianin C, Sanguiin H-6, and Punicalagin) incorrectly categorized as hydroxybenzoic acids in Phenol-Explorer, which molecular examination confirmed as ellagitannins. This classification discrepancy is consistent with previously documented challenges in polyphenol categorization [24,25], as noted by Neveu et al. [26] and Rothwell et al. [27]. Such misclassifications underscore the need for systematic structural verification when integrating data across databases and highlight the value of computational approaches in identifying taxonomic inconsistencies.

Drug-likeness evaluation using established computational metrics revealed complementary assessment patterns. The QED score distribution exhibited a distinctive bimodal pattern, indicating two predominant structural subclasses with differentiated physicochemical profiles. This bimodality likely reflects the inherent structural diversity within flavonoid and phenolic acid subclasses, particularly differences in glycosylation patterns and molecular complexity. The observation is consistent with principal component analyses reported in the recent literature [28], suggesting that these compound subclasses occupy distinct regions of chemical space with respect to pharmaceutical properties.

DataWarrior drug-likeness scores displayed a notably left-skewed distribution. Notably, 25% of compounds showed favorable drug-likeness scores above 0.05, identifying a substantial subset with promising pharmaceutical properties. Modified flavonoids demonstrated superior drug-likeness profiles, with sulfated and glucuronidated conjugates (e.g., 3′-O-Methyl-(-)-epicatechin 4′-O-sulfate, Genistein-7-O-glucuronide-4′-sulfate) achieving the highest scores. This suggests that specific phase II metabolite forms may possess improved computational drug-likeness scores compared to their parent aglycones, though experimental validation is required, an observation with implications for prodrug design and metabolite-based therapeutic strategies.

Correlation analysis between QED and DataWarrior scores revealed a moderate positive relationship, indicating that while both metrics evaluate drug-likeness, they capture complementary aspects of molecular properties through their distinct algorithmic approaches. QED demonstrated expected negative correlations with molecular weight, hydrogen bond donors, hydrogen bond acceptors, and topological polar surface area, consistent with its penalty function for excessive values in these parameters. The moderate correlation strengths suggest that the combined application of multiple drug-likeness metrics provides more comprehensive compound evaluation than single-metric approaches, supporting their complementary use in phytochemical assessment [29,30].

The moderate correlation between QED and DataWarrior scores reflects their distinct algorithmic foundations. QED integrates eight weighted physicochemical descriptors, penalizing deviations from optimal drug-like ranges [19], while DataWarrior evaluates substructure fragment frequencies derived from approved drugs [20]. Consequently, compounds may score favorably on one metric but not the other, depending on their structural features. Isoflavones demonstrate favorable scores on both metrics, whereas flavonols and anthocyanins show lower QED scores primarily due to higher molecular weights and hydrogen bonding capacity, features less penalized by DataWarrior’s fragment-based approach. For practical compound screening, we recommend a complementary dual-metric strategy: compounds ranking favorably on both QED and DataWarrior represent priority candidates for pharmaceutical development, while compounds scoring well on only one metric warrant case-by-case evaluation considering specific structural features and intended applications. This approach leverages the complementary strengths of both algorithms to minimize false negatives in drug-likeness assessment.

Compound evaluation across subclasses revealed isoflavones as demonstrating the most favorable drug-likeness profiles, with moderate molecular weight, acceptable LogP, and hydrogen bonding capacity within optimal ranges. In contrast, more complex subclasses such as flavan-3-ols and flavonols showed lower drug-likeness scores, primarily due to higher molecular weights, excessive hydrogen bond donors, and elevated polar surface areas. Top-performing individual compounds included sativanone, dihydroformononetin, and violanone, which warrant further investigation based on their favorable quantitative parameters and documented bioactivity [28,31]. The level of prior characterization among top-ranked compounds varies considerably. Diosmin (DataWarrior rank #4; DrugBank ID: DB08995) is approved in Europe and Asia for chronic venous insufficiency [32], while naringenin and liquiritigenin (QED ranks #10 and #9) are commercially available dietary supplements [33]. Quercetin and genistein are also extensively studied. In contrast, sativanone (QED rank #1), violanone (QED rank #3), and dihydroformononetin (QED rank #2) remain relatively underexplored despite favorable drug-likeness profiles. The concordance between computational drug-likeness predictions and real-world applications of several top-ranked compounds suggests that integrating multiple in silico metrics with molecular interaction and dietary source data may facilitate the identification of candidates for pharmaceutical development or dietary supplementation strategies.

Molecular interaction analysis identified statistically significant differences in binding affinities between flavonoids and phenolic acids. Flavonoids demonstrated interactions with a broader range of molecular targets (67 unique proteins versus 33 for phenolic acids), with preferential binding to DNA repair proteins (Bloom syndrome protein: 9.70) and hormone receptors (thyroid hormone receptor beta-1: 9.22). In contrast, phenolic acids exhibited selective interaction with membrane transporters (SLCO1B1 and SLCO1B3: 9.00), suggesting distinct biochemical functions [34,35,36,37]. This differential target selectivity has implications for their respective biological activities and potential therapeutic applications, with flavonoids showing broader predicted pharmacological potential based on target diversity and phenolic acids demonstrating more focused cellular transport modulation.

The differential target selectivity observed provides mechanistic insight into the distinct biological activities reported for these compound subclasses. Flavonoid interactions with DNA repair proteins, particularly the Bloom syndrome protein, align with their documented anti-tumor activities. The Bloom syndrome protein is a RecQ helicase essential for genomic stability, and its modulation has been implicated in cancer cell sensitization to DNA-damaging agents [38]. Similarly, flavonoid binding to thyroid hormone receptor beta-1 may contribute to their reported metabolic effects, as this receptor regulates lipid metabolism and thermogenesis [39]. The high interaction strength with MAP kinase ERK2 and c-Jun N-terminal kinase 1 further supports anti-inflammatory mechanisms, since these kinases mediate pro-inflammatory signaling cascades [40]. In contrast, the selective interaction of phenolic acids with membrane transporters SLCO1B1 and SLCO1B3 has distinct functional implications. These organic anion-transporting polypeptides mediate hepatic uptake of endogenous and exogenous compounds, suggesting that phenolic acids may modulate cellular absorption processes and potentially influence drug-nutrient interactions [41]. This transporter selectivity may also facilitate phenolic acid accumulation in hepatocytes, providing a mechanistic basis for their documented hepatoprotective effects [42].

The broader distribution of flavonoid interaction strengths indicates greater molecular promiscuity compared to phenolic acids. This characteristic carries both advantages and limitations. Multi-target engagement may be beneficial for complex diseases involving multiple dysregulated pathways, potentially explaining the pleiotropic health effects attributed to flavonoid-rich diets [43]. However, promiscuity also increases the probability of off-target effects and complicates mechanistic interpretation, as attributing biological outcomes to specific target interactions becomes challenging. Consequently, flavonoids may be better suited for preventive nutritional applications rather than precision therapeutics, while the more selective phenolic acid profile may offer advantages for targeted pharmacological interventions. Comparative analysis identified three shared targets between both compound classes (SLCO1B1, SLCO1B3, and p53); however, interaction patterns differed, with flavonoids showing higher binding affinities and more numerous compounds targeting these shared proteins. This suggests that while both compound classes can interact with similar molecular targets, their binding characteristics and target selectivity profiles remain distinct, potentially enabling complementary therapeutic applications.

Food source analysis mapped compounds across concentration ranges from 500 to 14,500 mg/kg, providing quantitative data for dietary source evaluation. Herbs and spices contained the highest total flavonoid content (45,516 mg/kg), followed by fruits (40,490 mg/kg) and teas (37,101 mg/kg) [44,45,46]. Evening primrose was identified as the highest individual source, containing 14,500 mg/kg, followed by mango at 9000 mg/kg and red wine at 7500 mg/kg. Compound distribution analysis identified specific accumulation patterns across food groups: herbs and spices contained high quercetin percentages (32.74%) across 49 compounds; fruits exhibited the highest compound diversity (62 unique flavonoids) with gallic acid predominating (22.23%); and beverages showed selective compound profiles with 2-phenylethanol comprising 95.04% of total flavonoids. These quantitative distribution data provide an empirical basis for dietary source selection to achieve specific flavonoid intake targets and inform nutritional intervention strategies [44,47]. Importantly, high phytochemical concentration does not equate to dietary relevance. Foods with lower absolute concentrations but higher consumption frequency (e.g., apples, tea) may contribute more substantially to total intake than concentrated but rarely consumed sources (e.g., evening primrose), underscoring the need to consider both concentration and dietary patterns. To illustrate practical applicability, consider prioritizing compounds for anti-inflammatory dietary intervention. The framework identifies isoflavones as optimal candidates based on favorable drug-likeness, documented NF-κB pathway interactions, and accessible dietary sources. Cross-referencing with food source data identifies soy products as concentrated isoflavone sources (36.91% of total flavonoids). Among specific compounds, naringenin and liquiritigenin combine high QED scores (>0.87) with anti-inflammatory target profiles, suggesting these as priority candidates for further investigation. This stepwise approach—integrating drug-likeness, target profiles, and dietary accessibility—demonstrates how the framework supports evidence-based compound prioritization.

Several limitations should be considered when interpreting these findings. First, drug-likeness predictions based on computational metrics require experimental validation through in vitro permeability assays and in vivo bioavailability studies [48,49,50]. The established metrics employed (QED, DataWarrior, Lipinski, Veber) were developed primarily for synthetic pharmaceuticals and may systematically underestimate the pharmaceutical potential of natural products. Classical rules such as Lipinski’s Ro5 were derived from oral drug libraries and do not account for alternative absorption mechanisms utilized by many flavonoids, including active transport via glucose transporters (SGLT1) and organic anion transporters [51]. Notably, natural products and their derivatives frequently violate Ro5 criteria yet demonstrate significant in vivo bioactivity [52]. In our dataset, 47% of compounds violated at least one Ro5 criterion, yet many of these possess well-documented biological activities, suggesting that drug-likeness metrics should be interpreted as initial screening tools rather than definitive exclusion criteria. QED scores are weighted toward oral drug optimization, potentially underestimating compounds absorbed via active transport mechanisms. DataWarrior’s fragment-based approach may penalize novel scaffolds underrepresented in approved drug databases. Additionally, ChEMBL activity data derive from heterogeneous assay conditions across laboratories, introducing variability that may affect cross-study comparisons. In addition, gut microbiota extensively metabolize flavonoids and phenolic acids through glycoside hydrolysis, C-ring cleavage, and dehydroxylation, generating metabolites with altered bioactivity profiles [53,54]. Inter-individual variation in microbiome composition may explain inconsistent clinical outcomes for flavonoid interventions [55]. Additionally, food processing substantially influences compound content and bioaccessibility: thermal processing may degrade heat-sensitive flavonoids while fermentation can hydrolyze glycosides to more bioavailable aglycones [56]. The food source concentrations reported herein represent raw or minimally processed foods; actual dietary exposure will vary with preparation methods. Second, molecular interaction data from ChEMBL represent in vitro binding affinities that may not directly translate to in vivo biological effects due to factors including bioavailability, tissue distribution, and metabolic conversion. Third, food source quantification relies on reported concentrations subject to variation from environmental, agricultural, and analytical factors [57,58], and actual dietary intake depends on food preparation methods and matrix effects that may influence compound stability and bioaccessibility. Future studies should prioritize experimental validation of solubility, permeability, and LogP for the highest-ranked candidates identified herein. Notably, for several top-ranked compounds (e.g., naringenin, quercetin), published experimental LogP values show good agreement with computed values, providing preliminary support for the predictive validity of the computational approach [59].

In conclusion, this integrative cross-database approach provides a systematic characterization framework for flavonoids and phenolic acids, combining established drug-likeness metrics, molecular interaction analysis, and dietary source mapping. The methodology bridges cheminformatics, molecular pharmacology, and food science to establish a comprehensive foundation for evaluating these bioactive compounds. The complementary nature of different drug-likeness metrics supports their combined application in compound assessment, while the molecular interaction and food source data provide context for understanding biological activity and dietary relevance. This analytical framework may be extended to other natural product classes, including terpenoids, alkaloids, and carotenoids, provided that sufficient structural data are available in compatible databases.

## Figures and Tables

**Table 1:** Drug-likeness Metrics and Physicochemical Parameters of Flavonoid and Phenolic Acid Subclasses. The table presents mean values for key physicochemical parameters and drug-likeness scores across different flavonoid and phenolic acid subclasses, sorted by QED score. Count indicates the number of compounds in each subclass.

Class	Count	QED Score	DataWarrior Score	MW	LogP	HBD	HBA	TPSA
Isoflavones	110	0.62 ± 0.18	0.42 ± 2.1	364.4	1.60	3.7	7.4	126.0
Flavanones	93	0.54 ± 0.21	−0.89 ± 3.2	493.5	0.25	5.8	11.0	190.9
Flavones	66	0.51 ± 0.22	−1.12 ± 3.8	444.1	0.85	5.1	10.1	167.7
Hydroxycinnamic acids	141	0.48 ± 0.24	−1.45 ± 4.1	409.4	1.22	4.6	7.8	147.2
Flavonols	130	0.42 ± 0.25	−2.87 ± 4.9	565.3	−0.95	8.3	14.4	242.4
Anthocyanins	129	0.38 ± 0.22	−3.21 ± 5.2	579.3	−0.15	8.8	13.3	235.4
Flavan-3-ols	20	0.35 ± 0.19	−4.12 ± 4.8	576.9	2.80	9.6	12.7	227.9

**Table 2:** Top 10 Compounds Ranked by QED and DataWarrior Drug-Likeness Metrics with Chemical Structures.

Rank	Compound—QED Score	Structure
1	Sativanone—0.940	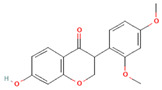
2	Dihydroformononetin—0.910	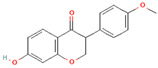
3	Violanone—0.900	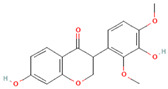
4	2,3-Dihydrobiochanin A—0.898	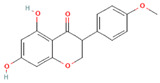
5	Isosakuranetin—0.895	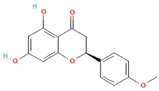
6	Vestitone—0.892	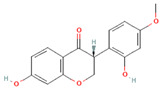
7	Sakuranetin—0.890	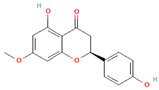
8	Dihydrobiochanin A—0.890	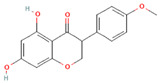
9	Liquiritigenin—0.885	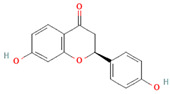
10	Naringenin—0.878	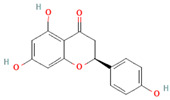
**Rank**	**Compound—DataWarrior Drug-Likeness Score**	**Structure**
1	3′-O-Methyl-(-)-epicatechin 4′-O-sulfate—2.648	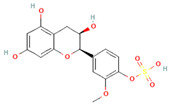
2	Genistein-7-O-glucuronide-4′-sulfate—2.562	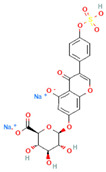
3	Kaempferol 3-O-(6″-acetyl-galactoside) 7-O-rhamnoside—2.456	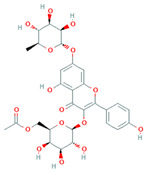
4	Diosmin—2.398	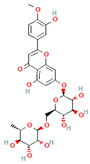
5	6-Hydroxyluteolin 7-O-rhamnoside—2.312	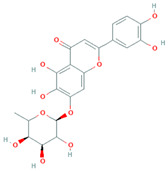
6	Ferulic acid 4-sulfate—2.287	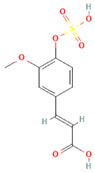
7	4′-O-Methyl-(-)-epicatechin 7-O-sulfate—2.245	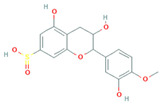
8	Homoplantaginin—2.198	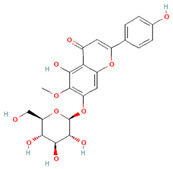
9	Hesperetin 3′-sulfate—2.156	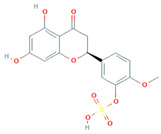
10	Kaempferol 3-O-glucuronide—2.102	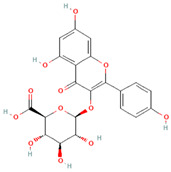

**Table 3:** Top Molecular Targets of Flavonoids and Phenolic Acids Ranked by ChEMBL Activity Score. Target proteins are ranked by mean ChEMBL activity score (−log M). ‘Count’ represents the number of distinct flavonoid compounds interacting with each target. ‘Mean’ indicates the average ChEMBL activity score across all interactions. ‘Max’ shows the highest activity score observed for any flavonoid-target pair. Higher scores indicate stronger binding affinity.

Target Name	Count	Mean	Max
Top Flavonoid Targets			
MAP kinase ERK2	1	9.00	9.00
c-Jun N-terminal kinase 1	1	9.00	9.00
Casein kinase I|	1	9.00	9.00
Thyroid hormone receptor beta-1	2	8.86	9.22
Solute carrier organic anion transporter family member 1B1	21	8.52	9.00
Canalicular multispecific organic anion transporter 1	5	8.44	9.00
Cyclooxygenase	2	8.34	8.34
Solute carrier organic anion transporter family member 1B3	30	8.32	9.00
Cellular tumor antigen p53	1	8.30	8.30
Bloom syndrome protein	2	8.00	9.70
Top Phenolic Acid Targets			
Solute carrier organic anion transporter family member 1B1	4	8.31	9.00
Cellular tumor antigen p53	1	8.20	8.20
Solute carrier organic anion transporter family member 1B3	6	8.04	9.00
Estrogen receptor alpha	1	7.93	7.93
CaM kinase II alpha	2	7.79	8.39
Muscarinic acetylcholine receptor M1	1	7.75	7.75
Nuclear factor NF-kappa-B p105 subunit	2	7.65	8.46
Matrix metalloproteinase-2	1	7.62	7.62
Androgen Receptor	1	7.40	7.40
Casein kinase II alpha, Casein kinase II, Casein kinase 2	1	7.12	7.12

## Data Availability

The databases investigated in this study are publicly available. The datasets analyzed during this study are provided as Supplementary Materials.

## Supplementary Materials

The following supporting information can be downloaded at: https://www.mdpi.com/article/10.3390/molecules31040728/s1.

## References

[1] Jubaidi F.F., Zainalabidin S., Taib I.S., Hamid Z.A., Budin S.B. (2021). The potential role of flavonoids in ameliorating diabetic cardiomyopathy via alleviation of cardiac oxidative stress, inflammation and apoptosis. Int. J. Mol. Sci..

[2] Zahra M., Abrahamse H., George B.P. (2024). Flavonoids: Antioxidant powerhouses and their role in nanomedicine. Antioxidants.

[3] Hasnat H., Shompa S.A., Islam M.M., Alam S., Richi F.T., Emon N.U., Ashrafi S., Ahmed N.U., Chowdhury M.N.R., Fatema N. (2024). Flavonoids: A treasure house of prospective pharmacological potentials. Heliyon.

[4] Ciumărnean L., Milaciu M.V., Runcan O., Vesa Ș.C., Răchișan A.L., Negrean V., Perné M.-G., Donca V.I., Alexescu T.-G., Para I. (2020). The effects of flavonoids in cardiovascular diseases. Molecules.

[5] Geng Q., Yan L., Shi C., Zhang L., Li L., Lu P., Cao Z., Li L., He X., Tan Y. (2024). Therapeutic effects of flavonoids on pulmonary fibrosis: A preclinical meta-analysis. Phytomedicine.

[6] Yuan D., Guo Y., Pu F., Yang C., Xiao X., Du H., He J., Lu S. (2024). Opportunities and challenges in enhancing the bioavailability and bioactivity of dietary flavonoids: A novel delivery system perspective. Food Chem..

[7] Kanakis C.D., Tarantilis P.A., Polissiou M.G., Diamantoglou S., Tajmir-Riahi H.A. (2007). An overview of DNA and RNA bindings to antioxidant flavonoids. Cell Biochem. Biophys..

[8] D’Arrigo G., Gianquinto E., Rossetti G., Cruciani G., Lorenzetti S., Spyrakis F. (2021). Binding of Androgen- and Estrogen-Like Flavonoids to Their Cognate (Non)Nuclear Receptors: A Comparison by Computational Prediction. Molecules.

[9] Wang L., Sweet D.H. (2012). Potential for food-drug interactions by dietary phenolic acids on human organic anion transporters 1 (SLC22A6), 3 (SLC22A8), and 4 (SLC22A11). Biochem. Pharmacol..

[10] Liu J., Du C., Beaman H.T., Monroe M.B.B. (2020). Characterization of Phenolic Acid Antimicrobial and Antioxidant Structure-Property Relationships. Pharmaceutics.

[11] Spagnuolo C., Russo M., Bilotto S., Tedesco I., Laratta B., Russo G.L. (2012). Dietary polyphenols in cancer prevention: The example of the flavonoid quercetin in leukemia. Ann. N. Y. Acad. Sci..

[12] Ye L.-S., Mu H.-F., Wang B.-L. (2025). Advances in flavonoid bioactivity in chronic diseases and bioavailability: Transporters and enzymes. J. Asian Nat. Prod. Res..

[13] Thilakarathna S.H., Rupasinghe H.P.V. (2013). Flavonoid bioavailability and attempts for bioavailability enhancement. Nutrients.

[14] Zhao J., Yang J., Xie Y. (2019). Improvement strategies for the oral bioavailability of poorly water-soluble flavonoids: An overview. Int. J. Pharm..

[15] Chow J., Yang X., Hu J., Yu Q., Zhong Y., Hu X., Liang J., Zhu C., Yan S., Li L. (2025). Gastrointestinal absorption and its regulation of hawthorn leaves flavonoids. Sci. Rep..

[16] Smeu A., Marcovici I., Dehelean C.A., Dumitrel S.-I., Borza C., Lighezan R. (2025). Flavonoids and Flavonoid-Based Nanopharmaceuticals as Promising Therapeutic Strategies for Colorectal Cancer-An Updated Literature Review. Pharmaceuticals.

[17] Stevens Barrón J.C., Chapa González C., Álvarez Parrilla E., De la Rosa L.A. (2023). Nanoparticle-Mediated Delivery of Flavonoids: Impact on Proinflammatory Cytokine Production: A Systematic Review. Biomolecules.

[18] Ertl P., Rohde B., Selzer P. (2000). Fast calculation of molecular polar surface area as a sum of fragment-based contributions and its application to the prediction of drug transport properties. J. Med. Chem..

[19] Bickerton G.R., Paolini G.V., Besnard J., Muresan S., Hopkins A.L. (2012). Quantifying the chemical beauty of drugs. Nat. Chem..

[20] Sander T., Freyss J., von Korff M., Rufener C. (2015). DataWarrior: An open-source program for chemistry aware data visualization and analysis. J. Chem. Inf. Model..

[21] openchemlib/src/main/resources/resources/druglikenessNoIndex.txt at Master Actelion/openchemlib. https://github.com/Actelion/openchemlib/blob/master/src/main/resources/resources/druglikenessNoIndex.txt.

[22] Lanuza F., Bondonno N.P., Zamora-Ros R., Rostgaard-Hansen A.L., Tjønneland A., Landberg R., Halkjær J., Andres-Lacueva C. (2022). Comparison of Flavonoid Intake Assessment Methods Using USDA and Phenol Explorer Databases: Subcohort Diet, Cancer and Health-Next Generations—MAX Study. Front. Nutr..

[23] Santos E.L., Maia B.H.L.N.S., Ferriani A.P., Teixeira S.D., Justino G.C. (2017). Flavonoids: Classification, biosynthesis and chemical ecology. Flavonoids—From Biosynthesis to Human Health.

[24] Al Mamari H.H. (2021). Phenolic compounds: Classification, chemistry, and updated techniques of analysis and synthesis. Phenolic Compounds—Chemistry, Synthesis, Diversity, Non-Conventional Industrial, Pharmaceutical and Therapeutic Applications.

[25] Kähkönen M., Kylli P., Ollilainen V., Salminen J.-P., Heinonen M. (2012). Antioxidant activity of isolated ellagitannins from red raspberries and cloudberries. J. Agric. Food Chem..

[26] Neveu V., Perez-Jiménez J., Vos F., Crespy V., du Chaffaut L., Mennen L., Knox C., Eisner R., Cruz J., Wishart D. (2010). Phenol-Explorer: An online comprehensive database on polyphenol contents in foods. Database.

[27] Rothwell J.A., Perez-Jimenez J., Neveu V., Medina-Remón A., M’hiri N., García-Lobato P., Manach C., Knox C., Eisner R., Wishart D.S. (2013). Phenol-Explorer 3.0: A major update of the Phenol-Explorer database to incorporate data on the effects of food processing on polyphenol content. Database.

[28] Baei B., Askari P., Askari F.S., Kiani S.J., Mohebbi A. (2025). Pharmacophore modeling and QSAR analysis of anti-HBV flavonols. PLoS ONE.

[29] Guardado Yordi E., Koelig R., Matos M.J., Pérez Martínez A., Caballero Y., Santana L., Pérez Quintana M., Molina E., Uriarte E. (2019). Artificial intelligence applied to flavonoid data in food matrices. Foods.

[30] Lee M.-H., Ta G.H., Weng C.-F., Leong M.K. (2020). In silico prediction of intestinal permeability by hierarchical support vector regression. Int. J. Mol. Sci..

[31] Hao B., Yang Z., Liu H., Liu Y., Wang S. (2024). Advances in flavonoid research: Sources, biological activities, and developmental prospectives. Curr. Issues Mol. Biol..

[32] Rahman L., Talha Khalil A., Ahsan Shahid S., Shinwari Z.K., Almarhoon Z.M., Alalmaie A., Sharifi-Rad J., Calina D. (2024). Diosmin: A promising phytochemical for functional foods, nutraceuticals and cancer therapy. Food Sci. Nutr..

[33] Saponara S., Fusi F., Iovinelli D., Ahmed A., Trezza A., Spiga O., Sgaragli G., Valoti M. (2021). Flavonoids and hERG channels: Friends or foes?. Eur. J. Pharmacol..

[34] Arango D., Morohashi K., Yilmaz A., Kuramochi K., Parihar A., Brahimaj B., Grotewold E., Doseff A.I. (2013). Molecular basis for the action of a dietary flavonoid revealed by the comprehensive identification of apigenin human targets. Proc. Natl. Acad. Sci. USA.

[35] Redan B.W., Buhman K.K., Novotny J.A., Ferruzzi M.G. (2016). Altered transport and metabolism of phenolic compounds in obesity and diabetes: Implications for functional food development and assessment. Adv. Nutr..

[36] Safe S., Jayaraman A., Chapkin R.S., Howard M., Mohankumar K., Shrestha R. (2021). Flavonoids: Structure-function and mechanisms of action and opportunities for drug development. Toxicol. Res..

[37] Liew Y.J.M., Lee Y.K., Khalid N., Rahman N.A., Tan B.C. (2020). Enhancing flavonoid production by promiscuous activity of prenyltransferase, BrPT2 from *Boesenbergia rotunda*. PeerJ.

[38] Tu J.-L., Wu B.-H., Wu H.-B., Wang J.-E., Zhang Z.-L., Gao K.-Y., Zhang L.-X., Chen Q.-R., Zhou Y.-C., Tan J.-H. (2023). Design, synthesis and evaluation of N3-substituted quinazolinone derivatives as potential Bloom’s Syndrome protein (BLM) helicase inhibitor for sensitization treatment of colorectal cancer. Eur. J. Med. Chem..

[39] Roth L., Hoffmann A., Hagemann T., Wagner L., Strehlau C., Sheikh B., Donndorf L., Ghosh A., Noé F., Wolfrum C. (2024). Thyroid hormones are required for thermogenesis of beige adipocytes induced by *Zfp423* inactivation. Cell Rep..

[40] Zhong R., Miao L., Zhang H., Tan L., Zhao Y., Tu Y., Angel Prieto M., Simal-Gandara J., Chen L., He C. (2022). Anti-inflammatory activity of flavonols via inhibiting MAPK and NF-κB signaling pathways in RAW264.7 macrophages. Curr. Res. Food Sci..

[41] Järvinen E., Deng F., Kiander W., Sinokki A., Kidron H., Sjöstedt N. (2021). The role of uptake and efflux transporters in the disposition of glucuronide and sulfate conjugates. Front. Pharmacol..

[42] Wu H., Zhang G., Huang L., Pang H., Zhang N., Chen Y., Wang G. (2017). Hepatoprotective Effect of Polyphenol-Enriched Fraction from Folium Microcos on Oxidative Stress and Apoptosis in Acetaminophen-Induced Liver Injury in Mice. Oxid. Med. Cell. Longev..

[43] Liu J., Li K., Yi Z., Saqirile, Wang C., Yang R. (2025). Oxidative-Inflammatory Crosstalk and Multi-Target Natural Agents: Decoding Diabetic Vascular Complications. Curr. Issues Mol. Biol..

[44] Waheed Janabi A.H., Kamboh A.A., Saeed M., Xiaoyu L., BiBi J., Majeed F., Naveed M., Mughal M.J., Korejo N.A., Kamboh R. (2020). Flavonoid-rich foods (FRF): A promising nutraceutical approach against lifespan-shortening diseases. Iran. J. Basic Med. Sci..

[45] Fecker R., Buda V., Alexa E., Avram S., Pavel I.Z., Muntean D., Cocan I., Watz C., Minda D., Dehelean C.A. (2020). Phytochemical and biological screening of *Oenothera biennis* L. hydroalcoholic extract. Biomolecules.

[46] Zhuang W.-B., Li Y.-H., Shu X.-C., Pu Y.-T., Wang X.-J., Wang T., Wang Z. (2023). The classification, molecular structure and biological biosynthesis of flavonoids, and their roles in biotic and abiotic stresses. Molecules.

[47] Panche A.N., Diwan A.D., Chandra S.R. (2016). Flavonoids: An overview. J. Nutr. Sci..

[48] Alruhaimi R.S., Mahmoud A.M., Alnasser S.M., Alotaibi M.F., Elbagory I., El-Bassuony A.A., Lamsabhi A.M., Kamel E.M. (2024). Integrating Computational Modeling and Experimental Validation to Unveil Tyrosinase Inhibition Mechanisms of Flavonoids from Alhagi graecorum. ACS Omega.

[49] Shrestha A., Marahatha R., Basnet S., Regmi B.P., Katuwal S., Dahal S.R., Sharma K.R., Adhikari A., Chandra Basnyat R., Parajuli N. (2022). Molecular Docking and Dynamics Simulation of Several Flavonoids Predict Cyanidin as an Effective Drug Candidate against SARS-CoV-2 Spike Protein. Adv. Pharmacol. Pharm. Sci..

[50] Tamil Selvan S., Ganta G.K. (2024). Computational investigations to identify potent natural flavonoid inhibitors of the nonstructural protein (NSP) 16/10 complex against coronavirus. Cureus.

[51] Miebs G., Mielniczuk A., Kadziński M., Bachorz R.A. (2024). Beyond the Arbitrariness of Drug-Likeness Rules: Rough Set Theory and Decision Rules in the Service of Drug Design. Appl. Sci..

[52] Benet L.Z., Hosey C.M., Ursu O., Oprea T.I. (2016). BDDCS, the Rule of 5 and drugability. Adv. Drug Deliv. Rev..

[53] Fiore M., Tonchev A.B., Pancheva R.Z., Yamashima T., Venditti S., Ferraguti G., Terracina S. (2025). Increasing Life Expectancy with Plant Polyphenols: Lessons from the Mediterranean and Japanese Diets. Molecules.

[54] Ozdal T., Sela D.A., Xiao J., Boyacioglu D., Chen F., Capanoglu E. (2016). The Reciprocal Interactions between Polyphenols and Gut Microbiota and Effects on Bioaccessibility. Nutrients.

[55] Healey G.R., Murphy R., Brough L., Butts C.A., Coad J. (2017). Interindividual variability in gut microbiota and host response to dietary interventions. Nutr. Rev..

[56] Toydemir G., Gultekin Subasi B., Hall R.D., Beekwilder J., Boyacioglu D., Capanoglu E. (2022). Effect of food processing on antioxidants, their bioavailability and potential relevance to human health. Food Chem. X.

[57] Sun C., Cao Y., Li X., Fang S., Yang W., Shang X. (2024). The impact of genetic similarity and environment on the flavonoids variation pattern of *Cyclocarya paliurus*. Sci. Rep..

[58] Chen J., Ning S., Lu X., Xiang W., Zhou X., Bu Y., Li L., Huang R. (2023). Variation in flavonoid and antioxidant activities of *Pyrrosia petiolosa* (Christ) Ching from different geographic origins. Front. Plant Sci..

[59] Sahani V., Patil S., Mahato A., Kumar M., Shanthi C.N. (2026). Molecular docking and physicochemical characterisation of kaempferol-hydroxy propyl-β-cyclodextrin–Inclusion complex. Chem. Phys. Impact.

